# Deep Sternal Wound Infection After Beating Heart Coronary Artery Bypass Surgery with Routine Use of Skeletonized Bilateral Internal Thoracic Artery

**DOI:** 10.21470/1678-9741-2021-0607

**Published:** 2023-06-14

**Authors:** Daniel M. S. Magalhães, Maurilio O. Deininger, Orlando Gomes de Oliveira, John Allexander de Freitas, Eugênia di Giuseppe Deininger

**Affiliations:** 1 Hospital Unimed João Pessoa - Alberto Urquiza Wanderley, João Pessoa, Paraíba, Brazil; 2 Hospital Memorial São Francisco, João Pessoa, Paraíba, Brazil

**Keywords:** Wound Infection, Angina, Unstable, Anti-Bacterial Agents, Coronary Artery Bypass, Off-Pump, Coronary Vessels

## Abstract

**Introduction:**

Despite its survival benefits, bilateral internal thoracic artery (BITA) grafting is not commonly utilized due to concerns over deep sternal wound infection (DSWI). We observed the role of routine use of BITA and off-pump coronary artery bypass grafting (OPCABG) in the incidence of DSWI and associated risk factors.

**Methods:**

Between January 2010 and December 2020, 1,207 patients were treated with isolated coronary artery bypass grafting. In all cases, OPCABG was attempted, and BITA was used whenever there was a need for a second arterial graft for the left coronary artery. DSWI was defined as a wound infection requiring surgical intervention and/or the administration of antibiotics. Multiple linear regression analysis was employed to model the risk of DSWI.

**Results:**

The incidence of DSWI was 0.58%. Mortality rate was higher in DSWI group than in no-DSWI group (28.57% vs. 1.25%; P<0.001). No significant difference in DSWI incidence was observed when BITA (70.6%) or single internal thoracic artery (29.4%) were used (P=0.680). The prevalence of diabetes (100% vs. 40.7%; P=0.001), hyperlipidemia (100% vs. 85.9%; P=0.045), and obesity (71.4% vs. 26.8%; P-0.017) was significantly elevated in DSWI group, when compared with no-DSWI group. Diabetes (P=0.0001), unstable angina (P=0.0064), previous myocardial infarction > 30 days (P=0.0009), left ventricular ejection fraction < 50% (P=0.0074), and emergency surgery (P=0.0002) were independent risk factors.

**Conclusion:**

The results of routine use of skeletonized BITA after OPCABG were satisfactory regarding DSWI incidence and operative mortality in a single-center experience.

**Table:** 

Abbreviations, Acronyms & Symbols	
BITA	= Bilateral internal thoracic artery
BMI	= Body mass index
CABG	= Coronary artery bypass grafting
COPD	= Chronic obstructive pulmonary disease
DSWI	= Deep sternal wound infection
ITA	= Internal thoracic artery
OPCABG	= Off-pump coronary artery bypass grafting
RITA	= Right internal thoracic artery
SITA	= Single internal thoracic artery

## INTRODUCTION

Deep sternal wound infection (DSWI) is a serious complication after coronary artery bypass grafting (CABG), which increases its morbidity and mortality. Several retrospective clinical trials have documented an increased risk of these complications associated with bilateral internal thoracic artery (BITA) harvesting, especially in patients with diabetes, chronic obstructive pulmonary disease (COPD), and obesity^[1]^.

The incidence of DSWI seems to be caused by the sternal ischemia that occurs after harvesting the BITA pedicle. Anatomical studies confirmed a decrease in sternal blood flow resulting from BITA dissection, and this was demonstrated by postoperative flow studies^[2]^. In order to minimize sternal complications, techniques such as skeletonized internal thoracic artery (ITA) dissection and low-energy electrocautery have been used^[3]^. Since these techniques were adopted, a reduction in wound infections has been reported when BITA grafts were removed, even in high-risk patients^[3,4,5]^.

Despite the demonstrated benefits of BITA over the single internal thoracic artery (SITA) in reintervention-free long-term survival, many surgeons still resist routine adoption of BITA grafting^[6,7]^. The technical challenge, the longer time spent, and the risk of infection are the main factors for restricting this technique to only 4-5% of CABG procedures worldwide^[8,9]^.

Some authors demonstrated that off-pump coronary artery bypass grafting (OPCABG), using skeletonized BITA, reduced postoperative complications, including wound infection, when compared to the conventional technique^[10,11]^.

We hypothesize that OPCABG with BITA can be used routinely even in high-risk patients, including those with diabetes, COPD, and obesity, despite the risk of DSWI. This study retrospectively analyzed, over 10 years, all patients undergoing CABG in a single center, to identify the incidence of DSWI and associated risk factors.

## METHODS

In this study, we performed a retrospective analysis of data from the hospital records of all patients undergoing isolated CABG in our service, in order to identify serious complications of the surgical wound. All patients were operated on by the same team of experienced surgeons.

The eligibility criterion was isolated CABG, therefore, patients undergoing combined procedures were excluded from this study. It was intended to perform OPCABG in all cases. From January 2010 to December 2020, a total of 2,211 patients underwent cardiac surgery, of which 1,207 (54.59%) were treated with isolated CABG.

### Graft Selection

It was based on the following strategies: 1) patients with severe multivessel disease (> 75%) involving the left coronary arteries or left main obstruction superior to 50% received BITA grafts; and 2) for minor lesions or to revascularize the right coronary artery, great saphenous veins grafts were used as additional conduits; ITA arteries were harvested in a skeletonized fashion with a conventional monopolar scalpel at low energy levels. The graft arrangement included: *in situ*, composite, or aortocoronary bypass. Distal anastomosis was generally performed with 8-0 polypropylene suture. The right internal thoracic artery (RITA) was most often anastomosed to the region of the left anterior descending artery, crossing anteriorly the aorta, and the left internal thoracic artery was usually anastomosed to the circumflex artery system in this set. For complete myocardial revascularization, saphenous vein or radial artery grafts were used as additional conduits.

### Perioperative Management

A few hours before surgery, the hairs around the surgical site were removed with clippers, and all patients were washed with a 2% chlorhexidine soap solution. In the operating room, traditional surgical scrubbing with a 2% chlorhexidine solution was performed. Before the skin incision, cefazolin (2 g) was initially administered, and additional doses (1 g) were administered every three hours during the procedure. The presternal space was closed with two layers of absorbable monofilament suture, followed by a continuous absorbable skin suture. Intravenous prophylactic antibiotics were administered after surgery for two days. During the procedure, blood glucose was routinely measured every two hours. An intravenous insulin infusion was given when blood glucose exceeded 150 mg/dL.

### Deep Sternal Wound Infection

DSWI was defined as a wound infection involving muscle, bone, and/or the mediastinum that meets any of the following conditions: open wound, with excision of tissue or re-exploration of mediastinum, positive culture, or treatment with antibiotics - based on the United States of America Centers for Disease Control guidelines -, and included patients who received required wound debridement and re-stitching^[12]^. For analysis purposes, all patients who were readmitted at any time in their evolution due to DSWI were considered.

### Statistical Analysis

Continuous variables are expressed as mean and standard deviation. The differences between groups were compared using Student’s *t*-test for normally distributed continuous data, or the Mann-Whitney U test for non-normally distributed continuous variables, and using the Fisher’s exact test for categorical variables. Multivariate analyses were performed by forward stepwise linear regression model. When dealing with continuous variables, in which the relationship with the outcome was not linear, we determined cutoff points, such as age, for example.

## RESULTS

### Preoperative Characteristics

A total of 1,207 patients were treated with isolated CABG, and only seven had DSWI (0.58%). Table 1 shows preoperative characteristics. The mean age was 72±6.8 years in DSWI *vs.* 65.14±9.9 years in no-DSWI group (*P*=0.067). The prevalence of diabetes mellitus (100% *vs.* 40.7%; *P*=0.001), hyperlipidemia (100% *vs.* 85.9%; *P*=0.045), and obesity (71.4% *vs.* 26.8%; *P*=0.017) were significant elevated in DSWI group, when compared with no-DSWI group.

**Table 1:** Demographic characteristics

Demographic Characteristics	All (N=1,207)	Deep wound sternal wound infection (N=7)	No deep sternal wound infection (N=1,200)	*P*-value
Age (years)	65.18 (±9.9)	72 (±6.8)	65.14 (±9.9)	0.067
Male	863 (71.5)	3 (42.9)	860 (71.7)	0.106
Unstable angina	241 (20.0)	3 (42.9)	238 (19.8)	0.146
New York Heart Association functional class - III/IV	386 (32.0)	2 (28.6)	384 (32.0)	0.846
Previous myocardial infarction > 30 days	628 (52.0)	1 (14.3)	627 (52.3)	0.059
Previous myocardial infarction < 30 days	97 (8.0)	1 (14.3)	96 (8.0)	0.542
Smoking	712 (59.0)	4 (57.1)	708 (59.0)	0.921
Diabetes	495 (41.0)	7 (100.0)	488 (40.7)	0.001
Hyperlipidemia	1,038 (86.0)	7 (100.0)	1,031 (85.9)	0.045
Obesity (BMI > 30)	326 (27.0)	5 (71.4)	321 (26.8)	0.017
Hypertension	809 (67.0)	4 (57.1)	805 (67.1)	0.690
Previous angioplasty	422 (35.0)	2 (28.6)	420 (35.0)	0.722
Left ventricular ejection fraction (30% - 40%)	36 (3.0)	0 (0.0)	36 (3.0)	0.643
Left ventricular ejection fraction (41% - 50%)	217 (18.0)	2 (28.6)	210 (17.5)	0.443
Left ventricular ejection fraction (> 50%)	954 (79.0)	5 (71.4)	957 (79.8)	0.583
Emergency surgery	241 (20.0)	2 (28.6)	231 (19.3)	0.358
Chronic obstructive lung disease	145 (12.0)	2 (28.6)	130 (10.8)	0.173

BMI=body mass index

### Operative Characteristics

Ninety-seven percent of surgeries were performed by OPCABG, with a conversion rate of 2.7% (Table 2). No significant difference was observed in DSWI incidence when surgery was converted from OPCABG (14.3% in DSWI group *vs.* 2.7% in no-DSWI group; *P*=0.209). No significant difference in DSWI incidence was observed when BITA (70.6%) or SITA (29.4%) were used (*P*=0.680). A no-touch aorta surgery was performed in 32.8%. In 65.1% of cases, RITA was used anteriorly crossing the aorta. The total number of distal anastomoses was 3,386 (mean of 2.8 anastomoses per patient). The operative mortality (in hospital) was significantly higher in patients with DSWI (28.57%) when compared with no-DSWI patients (1.25%; *P*<0.001).

**Table 2:** Surgical aspects.

Surgical Aspects	All (N=1,207)	Deep sternal wound infection (N=7)	No deep sternal wound infection (N=1,200)	*P*-value
OPCABG	1,174 (97.3)	6 (85.7)	1,168 (97.3)	0.180
Conversion from OPCABG	33 (2.7)	1 (14.3)	32 (2.7)	0.209
No-touch aorta	396 (32.8)	3 (42.9)	393 (32.8)	0.689
Bilateral internal thoracic artery	852 (70.6)	6 (85.7)	846 (70.5)	0.459
Single internal thoracic artery	355 (29.4)	1 (14.3)	354 (29.5)	0.459
Right internal thoracic artery crossover	786 (65.1)	6 (85.7)	778 (64.8)	0.685
Saphenous vein	934 (77.4)	4 (57.1)	930 (77.5)	0.525
Number of distal anastomoses	3,386 (2.8)	18 (2.6)	3,368 (2.8)	0.448
	64 (5.3)	0 (0,0)	64 (5.3)	
	330 (27.3)	3 (42.9)	227 (18.9)	
	612 (50.7)	4 (57.1)	608 (50.7)	
	201 (16.7)	0 (0,0)	201 (16.8)	
Operative mortality (in hospital)	17 (1.4)	2 (28.57)	15 (1.25)	< 0.001

OPCABG=off-pump coronary artery bypass grafting

### Risk

Linear regression analysis demonstrated that diabetes, unstable angina, previous myocardial infarction, left ventricular ejection fraction < 50%, and emergency surgery were strong independent risk factors for DSWI (Table 3).

**Table 3:** Linear regression analysis.

Characteristics	*P*-value
Age > 70 years	0.0963
Sex	0.1610
Unstable angina	0.0064
New York Heart Association functional class - III/IV	0.0627
Previous myocardial infarction > 30 days	0.0009
Previous myocardial infarction < 30 days	0.3792
Smoking	0.9099
Diabetes	0.0001
Hyperlipidemia	0.0746
Obesity (BMI > 30)	0.2768
Hypertension	0.0705
Previous angioplasty	0.1489
Left ventricular ejection fraction (30 - 40)	0.6979
Left ventricular ejection fraction (41 - 50)	0.0074
Left ventricular ejection fraction (> 50)	0.5830
Emergency surgery	0.0002
Chronic obstructive lung disease	0.0508

BMI=body mass index

## DISCUSSION

The present study using data from a single center showed satisfactory results of isolated OPCABG with a higher rate of BITA graft use (70.6%) and low incidence of DSWI (0.58%) and hospital mortality (1.4%).

The main reason why cardiovascular surgeons are hesitant to perform BITA grafts is DSWI, especially in patients with multiple preoperative risk factors such as diabetes mellitus, low ejection fraction, lung disease, and chronic kidney disease^[13-15]^. In fact, the present study showed that the rate of severe postoperative complications, including in-hospital mortality, was significantly higher in patients with DSWI (28.57%) compared to no-DSWI patients (1.25%) (*P*<0.001), demonstrating the severity of this complication. However, the survival benefits of the BITA graft for these high-risk patients have been also reported compared to the SITA graft surgery^[6,7]^. Therefore, a proper risk assessment of the DSWI is essential to minimize risk and maximize the long-term benefits derived from using BITA. The incidence of DSWI in the present cohort (0.58%) was acceptable compared to reports from various centers or national cohorts (1.4%-5.2%)^[4,15,16]^. The high prevalence of diabetes (41% in this cohort *vs.* 18.2%-33.3% in patients undergoing BITA graft in previous studies)^[6,8,9,17,18]^, previous percutaneous coronary intervention (35% *vs.* 3.2% -16.7 in patients undergoing BITA graft in previous studies)^[14,19,20]^, and the higher mean age (65.1±9.9 years *vs.* 56.0-60.1±5.5 years) are in agreement with the selection profile in which all patients were listed consecutively, better representing the “real-world” situation^[6,8,18,21]^. Regarding patients with diabetes, the incidence of DSWI receiving BITA grafts ranges from 2.9 to 11.6%^[4,15,20,22]^ and in the present study it was 1.71%, which confirms the low incidence of DSWI in this cohort, even in high risk patients. OPCABG was performed in 97.3% of patients in this study. It is expected that OPCABG reduces the systemic perioperative inflammatory response; however, OPCABG has not been identified as an independent factor to reduce DSWI^[17,20]^. Despite a low conversion rate from OPCABG to on-pump CABG (2.7%), no significant difference was observed in DSWI incidence when surgery was converted from OPCABG (14.3% in DSWI group *vs.* 2.7% in no-DSWI group; *P*=0.209). The absence of difference may be related to the small number of converted cases. A larger number of patients would be needed to identify whether conversion would be a risk factor for DSWI. A recent sub-analysis of the Arterial Revascularization Trial clearly demonstrated that a skeletonized BITA reduces the risk of DSWI compared to a pedicled BITA^[20]^. Even with a high rate use of BITA (70.6%) in this study, there was no significant increase in DSWI compared to similar studies^[14,18,20]^ or compared to SITA use (*P*=0.683), perhaps due to careful graft dissection by experienced surgeons, but this may not be applicable to surgeon training centers, for example. A lower incidence of DSWI in this cohort could be influenced by skeletonized use in all cases. The mean age was higher in DSWI group (72±6.8 years) *vs.* 65.14±9.9 years in no-DSWI group (*P*=0.067), despite not having reached a statistically significant difference. Perhaps a study with a greater number of cases could have shown a difference, since age is an additional risk factor for complications. The prevalence of diabetes mellitus (100% *vs.* 40.7%; *P*=0.001), hyperlipidemia (100% *vs.* 85.9%; *P*=0.045), and obesity (71.4% *vs.* 26.8%; *P*-0.017) were significant elevated in DSWI group, when compared with no-DSWI group. These findings are in agreement with some previous studies^[14,18,20]^. Often, obese diabetic patients are excluded from studies using BITA, due to the higher risk of infectious complications.

In the present risk model, diabetes mellitus has been recognized as the main risk factor for DSWI^[16-18,22]^. Preoperative hemodynamic status, such as an ejection fraction < 60%, is an independent risk factor for DSWI. These results are reasonable because a low output status has been recognized as a risk factor for infection^[17,20]^. Unstable angina and emergency surgery were independent risk factors too for DSWI in this study. These results may be in agreement with the greater severity of these patients, including the possible use of dual antiplatelet therapy at the time of surgery and a poor diabetes control, which can lead to longer surgical time and higher incidence of bleeding and infection.

### Limitations

We must note several limitations in this study. It was a retrospective study using a single-center hospital database with no mortality long-term results. The results of this study may be biased because it is a single center, with a relatively low volume of surgeries and with a unique team of dedicated and experienced surgeons, which may not represent the “real-world” in high-volume institutions. Therefore, the choice of patients who will undergo BITA must be done carefully in most centers.

## CONCLUSION

We reported the results and risks of DSWI after OPCABG BITA grafting from a single center; the results of routine use of BITA grafting were acceptable, and the incidence of hospital death after DSWI was higher than that with no-DSWI. The current data and risk model are informative to evaluate the risk of DSWI when performing OPCABG BITA grafting. Additional long-term prospective and multicenter studies are still needed to validate the routine use of BITA for all patients.

**Table:** 

Authors’ Roles & Responsibilities	
DMSM	Substantial contributions to the conception or design of the work; or the acquisition, analysis, or interpretation of data for the work; final approval of the version to be published
MOD	Substantial contributions to the conception or design of the work; or the acquisition, analysis, or interpretation of data for the work; final approval of the version to be published
OGO	Substantial contributions to the conception or design of the work; or the acquisition, analysis, or interpretation of data for the work; final approval of the version to be published
JAF	Substantial contributions to the conception or design of the work; or the acquisition, analysis, or interpretation of data for the work; final approval of the version to be published
EGD	Substantial contributions to the conception or design of the work; or the acquisition, analysis, or interpretation of data for the work; final approval of the version to be published
